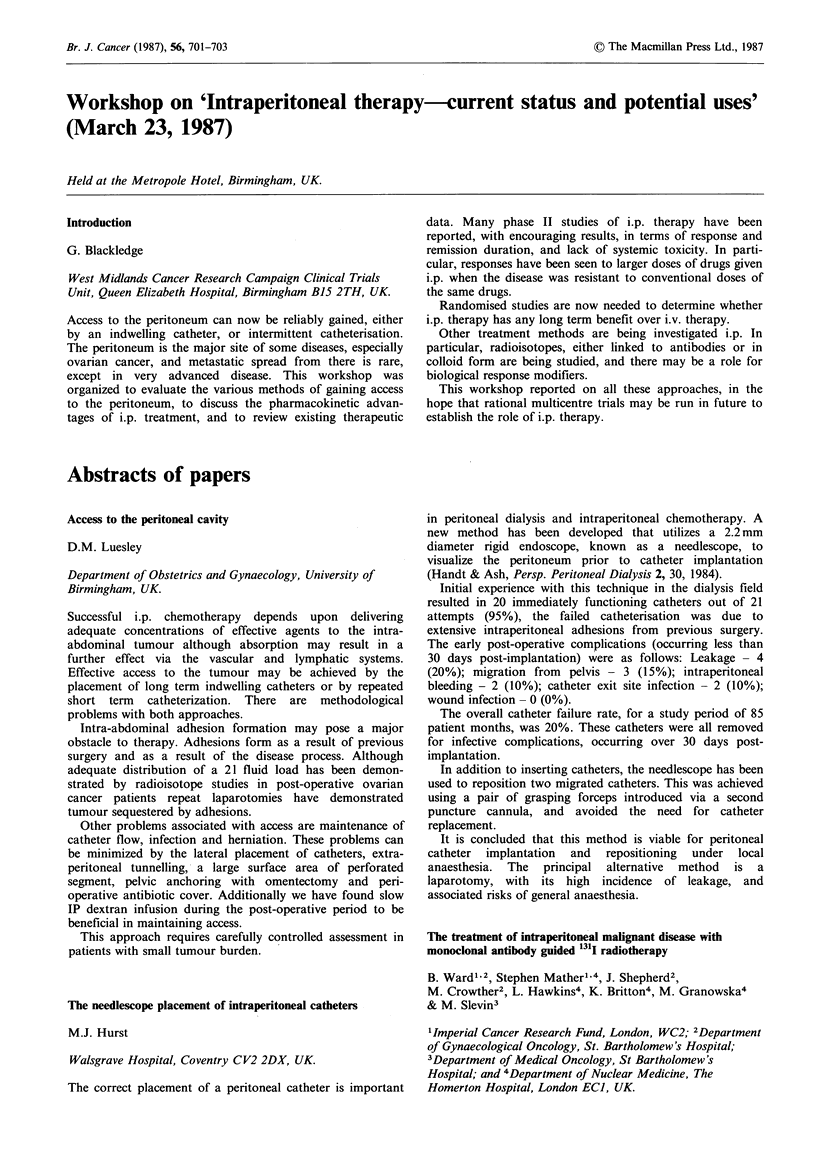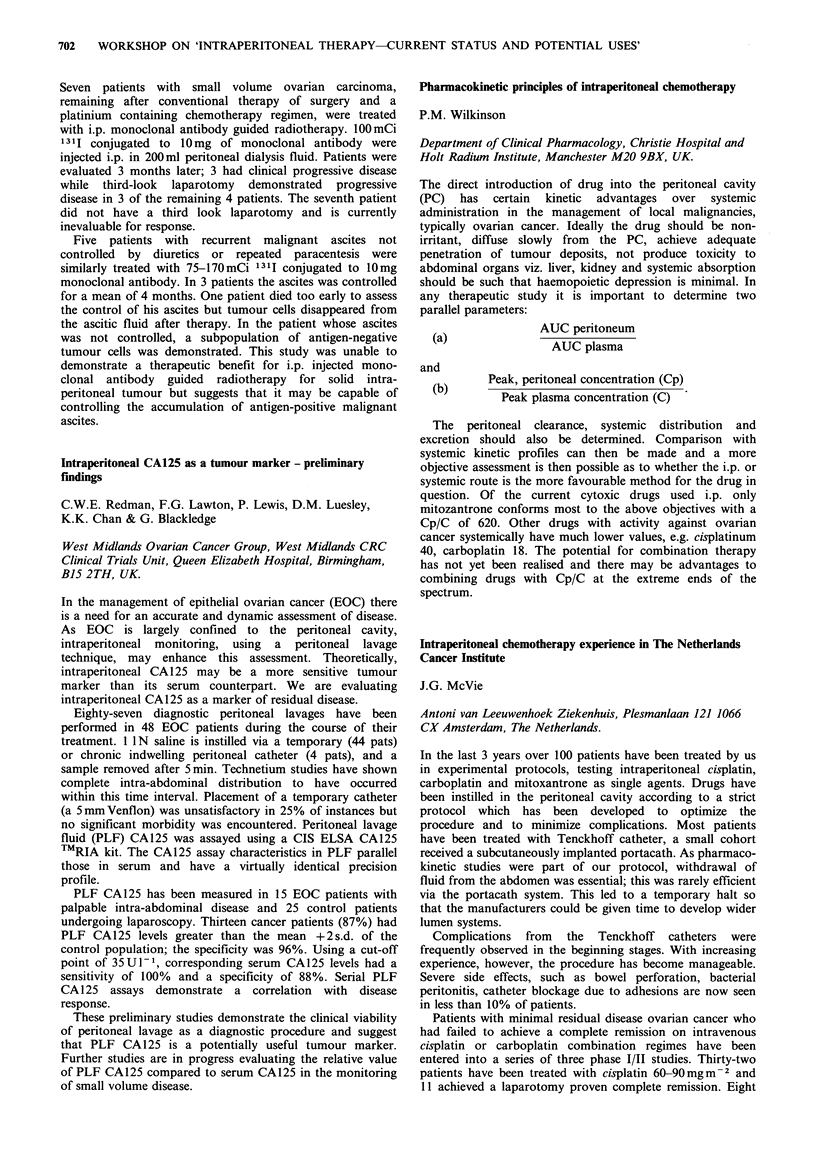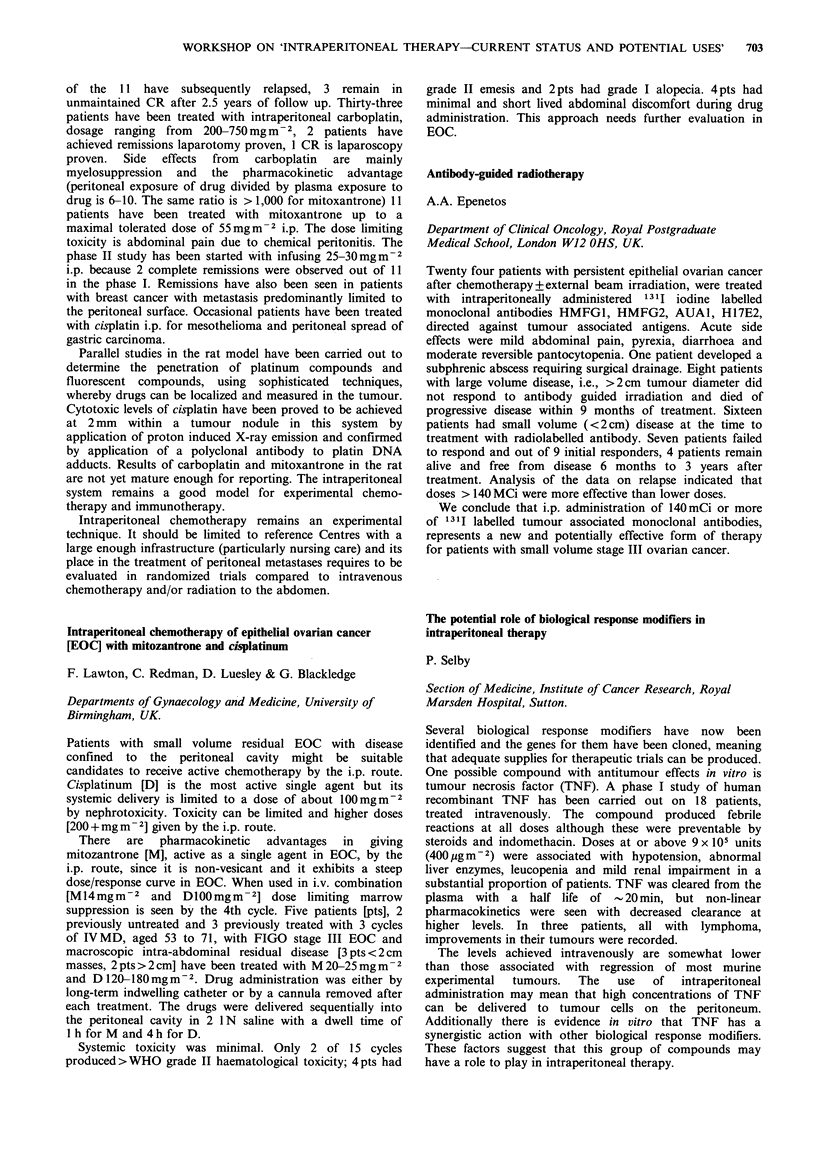# Workshop on 'Intraperitoneal therapy--current status and potential uses' (March 23, 1987). Birmingham, UK.Abstracts.

**Published:** 1987-11

**Authors:** 


Br. J. Cancer (1987), 56, 701–703 © The Macmillan Press Ltd., 1987

### Workshop on 'Intraperitoneal therapy—current status and potential uses' (March 23, 1987)

*Held at the Metropole Hotel, Birmingham, UK.*

**Introduction**

G. Blackledge

*West Midlands Cancer Research Campaign Clinical Trials Unit, Queen Elizabeth Hospital, Birmingham B15 2TH, UK.*

Access to the peritoneum can now be reliably gained, either by an indwelling catheter, or intermittent catheterisation. The peritoneum is the major site of some diseases, especially ovarian cancer, and metastatic spread from there is rare, except in very advanced disease. This workshop was organized to evaluate the various methods of gaining access to the peritoneum, to discuss the pharmacokinetic advantages of i.p. treatment, and to review existing therapeutic data. Many phase II studies of i.p. therapy have been reported, with encouraging results, in terms of response and remission duration, and lack of systemic toxicity. In particular, responses have been seen to larger doses of drugs given i.p. when the disease was resistant to conventional doses of the same drugs.

Randomised studies are now needed to determine whether i.p. therapy has any long term benefit over i.v. therapy.

Other treatment methods are being investigated i.p. In particular, radioisotopes, either linked to antibodies or in colloid form are being studied, and there may be a role for biological response modifiers.

This workshop reported on all these approaches, in the hope that rational multicentre trials may be run in future to establish the role of i.p. therapy.

### Abstracts of papers

**Access to the peritoneal cavity**

D.M. Luesley

*Department of Obstetrics and Gynaecology, University of Birmingham, UK.*

Successful i.p. chemotherapy depends upon delivering adequate concentrations of effective agents to the intra-abdominal tumour although absorption may result in a further effect via the vascular and lymphatic systems. Effective access to the tumour may be achieved by the placement of long term indwelling catheters or by repeated short term catheterization. There are methodological problems with both approaches.

Intra-abdominal adhesion formation may pose a major obstacle to therapy. Adhesions form as a result of previous surgery and as a result of the disease process. Although adequate distribution of a 2 l fluid load has been demonstrated by radioisotope studies in post-operative ovarian cancer patients repeat laparotomies have demonstrated tumour sequestered by adhesions.

Other problems associated with access are maintenance of catheter flow, infection and herniation. These problems can be minimized by the lateral placement of catheters, extra-peritoneal tunnelling, a large surface area of perforated segment, pelvic anchoring with omentectomy and peri-operative antibiotic cover. Additionally we have found slow IP dextran infusion during the post-operative period to be beneficial in maintaining access.

This approach requires carefully controlled assessment in patients with small tumour burden.

**The needlescope placement of intraperitoneal catheters**

M.J. Hurst

*Walsgrave Hospital, Coventry CV2 2DX, UK.*

The correct placement of a peritoneal catheter is important in peritoneal dialysis and intraperitoneal chemotherapy. A new method has been developed that utilizes a 2.2 mm diameter rigid endoscope, known as a needlescope, to visualize the peritoneum prior to catheter implantation (Handt & Ash, *Persp. Peritoneal Dialysis* **2**, 30, 1984).

Initial experience with this technique in the dialysis field resulted in 20 immediately functioning catheters out of 21 attempts (95%), the failed catheterisation was due to extensive intraperitoneal adhesions from previous surgery. The early post-operative complications (occurring less than 30 days post-implantation) were as follows: Leakage – 4 (20%); migration from pelvis – 3 (15%); intraperitoneal bleeding – 2 (10%); catheter exit site infection – 2 (10%); wound infection – 0 (0%).

The overall catheter failure rate, for a study period of 85 patient months, was 20%. These catheters were all removed for infective complications, occurring over 30 days post-implantation.

In addition to inserting catheters, the needlescope has been used to reposition two migrated catheters. This was achieved using a pair of grasping forceps introduced via a second puncture cannula, and avoided the need for catheter replacement.

It is concluded that this method is viable for peritoneal catheter implantation and repositioning under local anaesthesia. The principal alternative method is a laparotomy, with its high incidence of leakage, and associated risks of general anaesthesia.

**The treatment of intraperitoneal malignant disease with monoclonal antibody guided $^{131}I$ radiotherapy**

B. Ward[1,2], Stephen Mather[1,4], J. Shepherd[2], M. Crowther[2], L. Hawkins[4], K. Britton[4], M. Granowska[4] & M. Slevin[3]

[1]*Imperial Cancer Research Fund, London, WC2;* [2]*Department of Gynaecological Oncology, St. Bartholomew's Hospital;* [3]*Department of Medical Oncology, St Bartholomew's Hospital; and* [4]*Department of Nuclear Medicine, The Homerton Hospital, London EC1, UK.*

Seven patients with small volume ovarian carcinoma, remaining after conventional therapy of surgery and a platinium containing chemotherapy regimen, were treated with i.p. monoclonal antibody guided radiotherapy. 100 mCi $^{131}I$ conjugated to 10 mg of monoclonal antibody were injected i.p. in 200 ml peritoneal dialysis fluid. Patients were evaluated 3 months later; 3 had clinical progressive disease while third-look laparotomy demonstrated progressive disease in 3 of the remaining 4 patients. The seventh patient did not have a third look laparotomy and is currently inevaluable for response.

Five patients with recurrent malignant ascites not controlled by diuretics or repeated paracentesis were similarly treated with 75–170 mCi $^{131}I$ conjugated to 10 mg monoclonal antibody. In 3 patients the ascites was controlled for a mean of 4 months. One patient died too early to assess the control of his ascites but tumour cells disappeared from the ascitic fluid after therapy. In the patient whose ascites was not controlled, a subpopulation of antigen-negative tumour cells was demonstrated. This study was unable to demonstrate a therapeutic benefit for i.p. injected monoclonal antibody guided radiotherapy for solid intraperitoneal tumour but suggests that it may be capable of controlling the accumulation of antigen-positive malignant ascites.

### Intraperitoneal CA125 as a tumour marker – preliminary findings

C.W.E. Redman, F.G. Lawton, P. Lewis, D.M. Luesley, K.K. Chan & G. Blackledge

*West Midlands Ovarian Cancer Group, West Midlands CRC Clinical Trials Unit, Queen Elizabeth Hospital, Birmingham, B15 2TH, UK.*

In the management of epithelial ovarian cancer (EOC) there is a need for an accurate and dynamic assessment of disease. As EOC is largely confined to the peritoneal cavity, intraperitoneal monitoring, using a peritoneal lavage technique, may enhance this assessment. Theoretically, intraperitoneal CA125 may be a more sensitive tumour marker than its serum counterpart. We are evaluating intraperitoneal CA125 as a marker of residual disease.

Eighty-seven diagnostic peritoneal lavages have been performed in 48 EOC patients during the course of their treatment. 1 l N saline is instilled via a temporary (44 pats) or chronic indwelling peritoneal catheter (4 pats), and a sample removed after 5 min. Technetium studies have shown complete intra-abdominal distribution to have occurred within this time interval. Placement of a temporary catheter (a 5 mm Venflon) was unsatisfactory in 25% of instances but no significant morbidity was encountered. Peritoneal lavage fluid (PLF) CA125 was assayed using a CIS ELSA CA125 ™RIA kit. The CA125 assay characteristics in PLF parallel those in serum and have a virtually identical precision profile.

PLF CA125 has been measured in 15 EOC patients with palpable intra-abdominal disease and 25 control patients undergoing laparoscopy. Thirteen cancer patients (87%) had PLF CA125 levels greater than the mean +2 s.d. of the control population; the specificity was 96%. Using a cut-off point of 35 U $l^{-1}$, corresponding serum CA125 levels had a sensitivity of 100% and a specificity of 88%. Serial PLF CA125 assays demonstrate a correlation with disease response.

These preliminary studies demonstrate the clinical viability of peritoneal lavage as a diagnostic procedure and suggest that PLF CA125 is a potentially useful tumour marker. Further studies are in progress evaluating the relative value of PLF CA125 compared to serum CA125 in the monitoring of small volume disease.

### Pharmacokinetic principles of intraperitoneal chemotherapy

P.M. Wilkinson

*Department of Clinical Pharmacology, Christie Hospital and Holt Radium Institute, Manchester M20 9BX, UK.*

The direct introduction of drug into the peritoneal cavity (PC) has certain kinetic advantages over systemic administration in the management of local malignancies, typically ovarian cancer. Ideally the drug should be non-irritant, diffuse slowly from the PC, achieve adequate penetration of tumour deposits, not produce toxicity to abdominal organs viz. liver, kidney and systemic absorption should be such that haemopoietic depression is minimal. In any therapeutic study it is important to determine two parallel parameters:

$$\text{(a)} \quad \frac{\text{AUC peritoneum}}{\text{AUC plasma}}$$

and

$$\text{(b)} \quad \frac{\text{Peak, peritoneal concentration (Cp)}}{\text{Peak plasma concentration (C)}}.$$

The peritoneal clearance, systemic distribution and excretion should also be determined. Comparison with systemic kinetic profiles can then be made and a more objective assessment is then possible as to whether the i.p. or systemic route is the more favourable method for the drug in question. Of the current cytoxic drugs used i.p. only mitozantrone conforms most to the above objectives with a Cp/C of 620. Other drugs with activity against ovarian cancer systemically have much lower values, e.g. *cis*platinum 40, carboplatin 18. The potential for combination therapy has not yet been realised and there may be advantages to combining drugs with Cp/C at the extreme ends of the spectrum.

### Intraperitoneal chemotherapy experience in The Netherlands Cancer Institute

J.G. McVie

*Antoni van Leeuwenhoek Ziekenhuis, Plesmanlaan 121 1066 CX Amsterdam, The Netherlands.*

In the last 3 years over 100 patients have been treated by us in experimental protocols, testing intraperitoneal *cis*platin, carboplatin and mitoxantrone as single agents. Drugs have been instilled in the peritoneal cavity according to a strict protocol which has been developed to optimize the procedure and to minimize complications. Most patients have been treated with Tenckhoff catheter, a small cohort received a subcutaneously implanted portacath. As pharmacokinetic studies were part of our protocol, withdrawal of fluid from the abdomen was essential; this was rarely efficient via the portacath system. This led to a temporary halt so that the manufacturers could be given time to develop wider lumen systems.

Complications from the Tenckhoff catheters were frequently observed in the beginning stages. With increasing experience, however, the procedure has become manageable. Severe side effects, such as bowel perforation, bacterial peritonitis, catheter blockage due to adhesions are now seen in less than 10% of patients.

Patients with minimal residual disease ovarian cancer who had failed to achieve a complete remission on intravenous *cis*platin or carboplatin combination regimes have been entered into a series of three phase I/II studies. Thirty-two patients have been treated with *cis*platin 60–90 mg $m^{-2}$ and 11 achieved a laparotomy proven complete remission. Eight of the 11 have subsequently relapsed, 3 remain in unmaintained CR after 2.5 years of follow up. Thirty-three patients have been treated with intraperitoneal carboplatin, dosage ranging from 200–750 $mg\,m^{-2}$, 2 patients have achieved remissions laparotomy proven, 1 CR is laparoscopy proven. Side effects from carboplatin are mainly myelosuppression and the pharmacokinetic advantage (peritoneal exposure of drug divided by plasma exposure to drug is 6–10. The same ratio is >1,000 for mitoxantrone) 11 patients have been treated with mitoxantrone up to a maximal tolerated dose of 55 $mg\,m^{-2}$ i.p. The dose limiting toxicity is abdominal pain due to chemical peritonitis. The phase II study has been started with infusing 25–30 $mg\,m^{-2}$ i.p. because 2 complete remissions were observed out of 11 in the phase I. Remissions have also been seen in patients with breast cancer with metastasis predominantly limited to the peritoneal surface. Occasional patients have been treated with *cis*platin i.p. for mesothelioma and peritoneal spread of gastric carcinoma.

Parallel studies in the rat model have been carried out to determine the penetration of platinum compounds and fluorescent compounds, using sophisticated techniques, whereby drugs can be localized and measured in the tumour. Cytotoxic levels of *cis*platin have been proved to be achieved at 2 mm within a tumour nodule in this system by application of proton induced X-ray emission and confirmed by application of a polyclonal antibody to platin DNA adducts. Results of carboplatin and mitoxantrone in the rat are not yet mature enough for reporting. The intraperitoneal system remains a good model for experimental chemotherapy and immunotherapy.

Intraperitoneal chemotherapy remains an experimental technique. It should be limited to reference Centres with a large enough infrastructure (particularly nursing care) and its place in the treatment of peritoneal metastases requires to be evaluated in randomized trials compared to intravenous chemotherapy and/or radiation to the abdomen.

### Intraperitoneal chemotherapy of epithelial ovarian cancer [EOC] with mitozantrone and *cis*platinum

F. Lawton, C. Redman, D. Luesley & G. Blackledge

*Departments of Gynaecology and Medicine, University of Birmingham, UK.*

Patients with small volume residual EOC with disease confined to the peritoneal cavity might be suitable candidates to receive active chemotherapy by the i.p. route. *Cis*platinum [D] is the most active single agent but its systemic delivery is limited to a dose of about 100 $mg\,m^{-2}$ by nephrotoxicity. Toxicity can be limited and higher doses [200 + $mg\,m^{-2}$] given by the i.p. route.

There are pharmacokinetic advantages in giving mitozantrone [M], active as a single agent in EOC, by the i.p. route, since it is non-vesicant and it exhibits a steep dose/response curve in EOC. When used in i.v. combination [M14 $mg\,m^{-2}$ and D100 $mg\,m^{-2}$] dose limiting marrow suppression is seen by the 4th cycle. Five patients [pts], 2 previously untreated and 3 previously treated with 3 cycles of IVMD, aged 53 to 71, with FIGO stage III EOC and macroscopic intra-abdominal residual disease [3 pts < 2 cm masses, 2 pts > 2 cm] have been treated with M 20–25 $mg\,m^{-2}$ and D 120–180 $mg\,m^{-2}$. Drug administration was either by long-term indwelling catheter or by a cannula removed after each treatment. The drugs were delivered sequentially into the peritoneal cavity in 2 1N saline with a dwell time of 1 h for M and 4 h for D.

Systemic toxicity was minimal. Only 2 of 15 cycles produced > WHO grade II haematological toxicity; 4 pts had grade II emesis and 2 pts had grade I alopecia. 4 pts had minimal and short lived abdominal discomfort during drug administration. This approach needs further evaluation in EOC.

### Antibody-guided radiotherapy

A.A. Epenetos

*Department of Clinical Oncology, Royal Postgraduate Medical School, London W12 0HS, UK.*

Twenty four patients with persistent epithelial ovarian cancer after chemotherapy ± external beam irradiation, were treated with intraperitoneally administered $^{131}I$ iodine labelled monoclonal antibodies HMFG1, HMFG2, AUA1, H17E2, directed against tumour associated antigens. Acute side effects were mild abdominal pain, pyrexia, diarrhoea and moderate reversible pantocytopenia. One patient developed a subphrenic abscess requiring surgical drainage. Eight patients with large volume disease, i.e., > 2 cm tumour diameter did not respond to antibody guided irradiation and died of progressive disease within 9 months of treatment. Sixteen patients had small volume (< 2 cm) disease at the time to treatment with radiolabelled antibody. Seven patients failed to respond and out of 9 initial responders, 4 patients remain alive and free from disease 6 months to 3 years after treatment. Analysis of the data on relapse indicated that doses > 140 MCi were more effective than lower doses.

We conclude that i.p. administration of 140 mCi or more of $^{131}I$ labelled tumour associated monoclonal antibodies, represents a new and potentially effective form of therapy for patients with small volume stage III ovarian cancer.

### The potential role of biological response modifiers in intraperitoneal therapy

P. Selby

*Section of Medicine, Institute of Cancer Research, Royal Marsden Hospital, Sutton.*

Several biological response modifiers have now been identified and the genes for them have been cloned, meaning that adequate supplies for therapeutic trials can be produced. One possible compound with antitumour effects *in vitro* is tumour necrosis factor (TNF). A phase I study of human recombinant TNF has been carried out on 18 patients, treated intravenously. The compound produced febrile reactions at all doses although these were preventable by steroids and indomethacin. Doses at or above $9 \times 10^5$ units (400 $\mu g\,m^{-2}$) were associated with hypotension, abnormal liver enzymes, leucopenia and mild renal impairment in a substantial proportion of patients. TNF was cleared from the plasma with a half life of ~20 min, but non-linear pharmacokinetics were seen with decreased clearance at higher levels. In three patients, all with lymphoma, improvements in their tumours were recorded.

The levels achieved intravenously are somewhat lower than those associated with regression of most murine experimental tumours. The use of intraperitoneal administration may mean that high concentrations of TNF can be delivered to tumour cells on the peritoneum. Additionally there is evidence *in vitro* that TNF has a synergistic action with other biological response modifiers. These factors suggest that this group of compounds may have a role to play in intraperitoneal therapy.